# Bibliometric Analysis of Worldwide Publications on Antimalarial Drug Resistance (2006–2015)

**DOI:** 10.1155/2017/6429410

**Published:** 2017-08-10

**Authors:** Waleed M. Sweileh, Samah W. Al-Jabi, Ansam F. Sawalha, Adham S. AbuTaha, Sa'ed H. Zyoud

**Affiliations:** ^1^Department of Physiology, Pharmacology and Toxicology, College of Medicine and Health Sciences, An-Najah National University, Nablus, State of Palestine; ^2^Department of Clinical and Community Pharmacy, College of Medicine and Health Sciences, An-Najah National University, Nablus, State of Palestine

## Abstract

**Background:**

In response to international efforts to control and eradicate malaria, we designed this study to give a bibliometric overview of research productivity in antimalarial drug resistance (AMDR).

**Methods:**

Keywords related to AMDR were used to retrieve relevant literature using Scopus database.

**Results:**

A total of 976 publications with an h-index of 63 were retrieved. The number of publications showed a noticeable increase starting in the early 1990s. The USA was the most productive country with 337 publications equivalent to one-third of worldwide publications in this field. More than two-thirds of publications by the USA (236, 70.03%) were made by international collaboration. Of the top ten productive countries, two countries were from Mekong subregion, particularly Thailand and Cambodia. The Malaria Journal was the most productive journal (136, 13.93%) in this field. Mahidol University (80, 8.20%) in Thailand was the most productive institution. Seven articles in the top-ten list were about artemisinin resistance in* Plasmodium falciparum*, one was about chloroquine resistance, one was about sulfadoxine-pyrimethamine resistance, and the remaining one was about general multidrug resistance.

**Conclusion:**

Eradication and control of AMDR require continuing research activity to help international health organizations identify spots that require an immediate action to implement appropriate measures.

## 1. Background

Malaria is a common and fatal infectious parasitic disease [1, 2]. It is transmitted through* Anopheles* mosquitoes [3–6]. It was estimated that 214 million new malaria cases occurred worldwide in 2015 [2]. Malaria control and eradication are one of the major goals of the United Nation's Millennium Development Goals (MDG). In goal number 6, target 6C, the MDG aimed to halt by half and reverse the incidence of malaria by 2015 [7]. This goal was successfully achieved when the World Health Organization (WHO) reported that, between 2000 and 2015, malaria incidence rates and mortality rates fell significantly in “Africa, Southeast Asia (SEA) regions, Western Pacific region, Eastern Mediterranean region,” and other regions in the world [8]. Vector control through insecticide-treated mosquito nets (ITNs) and indoor residual spraying (IRS) has contributed to the control and eradication of malaria in different world regions particularly in Africa [9–12]. Furthermore, the discovery of the effective drug artemisinin has greatly changed the therapeutic approach of malaria and enhanced control and eradication of malaria [13–15]. Artemisinin is isolated from the plant* Artemisia annua* employed in Chinese traditional medicine [16]. Actually, the Chinese scientist Tu Youyou, who discovered the drug artemisinin, was awarded Nobel Prize in Medicine in 2015 [17, 18]. Emergence of antimalarial drug resistance (AMDR), particularly for the core compound, artemisinin, is a new challenge for future plans to control malaria. In this regard, AMDR is defined as survival and multiplication of malaria parasite under conditions that normally stop and cure malaria infection [19]. One of the main advances in AMDR is the identification of mutations responsible for drug resistance [20–22]. Monitoring of AMDR is highly needed in order to adopt different control and therapeutic policies for malaria. Assessing research productivity on malaria in general and those pertaining to drug resistance in particular is extremely important. Such studies are carried out using bibliometric indicators that help identify research trends, hot research topics, international collaboration, and country contribution to the field. In fact several studies have been carried out using bibliometric indicators to assess malaria research in different parts of the world [23–29]. However, none was carried out on AMDR. Therefore, the aim of this study was to give a bibliometric overview of publications on AMDR. The focus of this study will be on documents published in the last decade (2006 to 2015) to give an insight into the most recent research activity in this field and future prospects in order to help health policy makers make future plans on malaria control more relevant.

## 2. Methods

The method and indicators used in this study have been explained in detail in previously published bibliometric studies [30–38]. However, we will present and discuss the approach used in this study as an additional piece of information for readers and other investigators. Scopus, run by Elsevier, is one of the largest electronic databases available for literature retrieval. It is friendly to use and provides functions like “limit” and “exclude” that facilitates data refining and analysis. Furthermore, Scopus has the ability to provide researchers with citation analysis, country profile, institution profile, author profile, and source journals for any set of data in any particular field. Other databases can be used for data analysis and retrieval; however, Scopus remains superior to these databases in terms of volume of literature it has [39].

In this study, the keywords used in Scopus for retrieval of data were as follows: (TITLE(“Plasmodium falciparum” OR “PLASMODIUM vivax” OR “Plasmodium malariae” OR “Plasmodium ovale” OR malaria OR “P. vivax” OR “P. falciparum” OR “P. malriae” OR “P. ovale”) AND TITLE(“ ^*∗*^aminoquinoline resist^*∗*^” OR “ ^*∗*^chloroquine resist^*∗*^” OR “amodiaquine resist^*∗*^” OR “pyrimethamine resist^*∗*^” OR “mefloquine resist^*∗*^” OR “artemisinin resist^*∗*^” OR “piperaquine resist^*∗*^” OR “resist^*∗*^ malaria” OR “antimalarial drug resist^*∗*^”) OR TITLE(“proguanil resist^*∗*^” OR “sulf^*∗*^resist^*∗*^” OR “Atovaquone resist^*∗*^” OR “Primaquine resist^*∗*^” OR “Halofantrine resist^*∗*^” OR pfcrt^*∗*^ OR pfmdr^*∗*^ OR pfatp^*∗*^ OR pfnhe^*∗*^ OR “dhfr^*∗*^ mutation” OR “dhps^*∗*^ mutation” OR pfmrp OR pfdhfr OR pfdhps) OR TITLE(pfmrp^*∗*^ OR pfcytb^*∗*^ OR “Chloroguanide resist^*∗*^” OR “quinine resist^*∗*^” OR “Pyronaridine resist^*∗*^” OR “dihydroartem^*∗*^  resist^*∗*^” OR “arte^*∗*^  resist^*∗*^” OR “drug resist^*∗*^ malaria” OR “resist^*∗*^” OR pvcrt^*∗*^ OR pvmdr^*∗*^) AND TITLE(resist^*∗*^) AND NOT TITLE(insect^*∗*^ OR anopheles OR tuberculosis OR pyrethroid OR mosquito OR avian OR toxoplasma OR cytochrome OR salmonella OR fluoroquinolone OR antifungal OR snake OR organophosphate)) AND PUBYEAR > 2005 AND PUBYEAR < 2016 AND (LIMIT-TO(SRCTYPE, “j”)) AND (EXCLUDE(DOCTYPE, “er”)).

These keywords used in this study were chosen based on literature review pertaining to AMDR from all aspects including molecular biology and genetics. To maximize accuracy, all keywords were entered in title search and quotation marks were used wherever appropriate. The time limit of the study was from 2006 to 2015. For the purpose of this study, only journal articles were included in the analysis. Quantitative assessment of AMDR literature was simply carried out by analysis of volume of retrieved articles while scientific impact of the publication was presented as number of citations per article and number of highly cited articles as well as the impact factor (IF) of journals publishing the retrieved articles. The validity of our search query was tested and confirmed by manually reviewing 10% of top cited articles in the retrieved data. The manual review was carried by the authors themselves. Country affiliation analysis in Scopus can give researchers insight into intra- and intercountry collaboration. Single country publications (SCP) are those that represent intracountry collaboration while multiple country publications (MCP) are those that represent intercountry collaboration. We considered only the top ten ranking countries, institutions, and journals. To visualize country collaboration or coauthorships, VOSviewer was used [40]. VOSviewer can represent information as either density visualizations maps or network visualizations maps. In this study, we used density visualization map as cluster density maps. Each cluster represents group of most frequently and closely collaborating countries where countries having higher numbers of coauthorships are the ones with higher extent of collaboration.

## 3. Results

A total of 976 journal documents were retrieved. Types of retrieved documents are listed in Table 1. Original research articles (790; 80.94%) were the main type. A total of 12 different languages were encountered in the retrieved documents. English language (942; 96.52%) was most commonly encountered followed by French (16; 1.64%) and Chinese (6; 0.30%) languages. A total of 125 countries contributed to the publication of retrieved documents.

**Figure 1:**
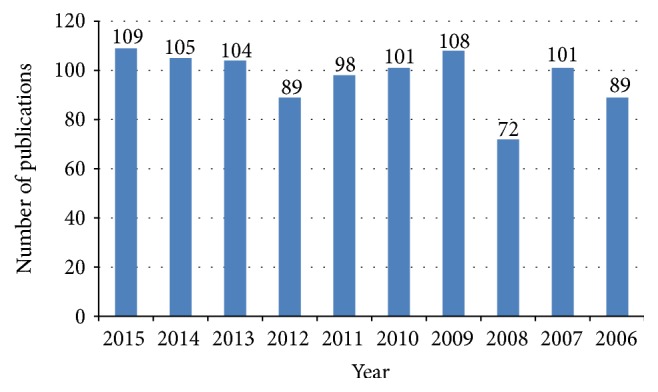
Growth of publications on AMDR (2006–2015).

**Figure 2:**
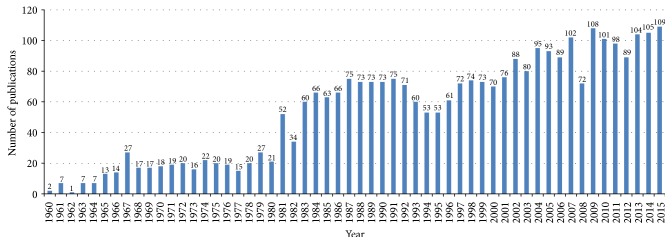
Growth of publications on AMDR (1960–2015).

**Figure 3:**
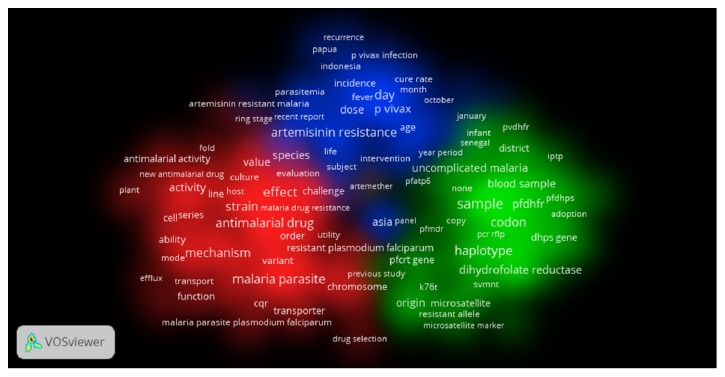
Cluster density visualization map of frequently encountered terms in title/abstract of retrieved documents on AMDR (2006–2015). A minimum of 10 yielded 350 terms.

The growth of publications on AMDR showed a fluctuating pattern in the last decade (Figure 1). However, growth of publications showed a noticeable increase when data on AMDR was presented for the last five decades (Figure 2). The average number of publications was approximately 98 documents per year.  Table 2 shows the number of publications, total citations, and average number of citations per article in each year for the last decade. The total number of citations of the retrieved documents was 21399 with an h-index of 63. VOSviewer technique was used to find out the most commonly encountered terms in title/abstract of retrieved documents after setting the minimum threshold at 10. The density visualization map yielded a total of 350 relevant terms distributed in three clusters shown in three different colors (Figure 3). Cluster number one (red) focuses on terms mainly related to chloroquine resistance (CQR) and the genetic basis behind CQR. The second cluster (green) focuses on antifolate drug resistance and the genetic basis of this resistance. The third cluster (blue) focuses on artemisinin related resistance and its geographical distribution in Asia and Africa.  Table 3 lists the most frequent terms in each cluster and the number of occurrences of each term.

**Figure 4:**
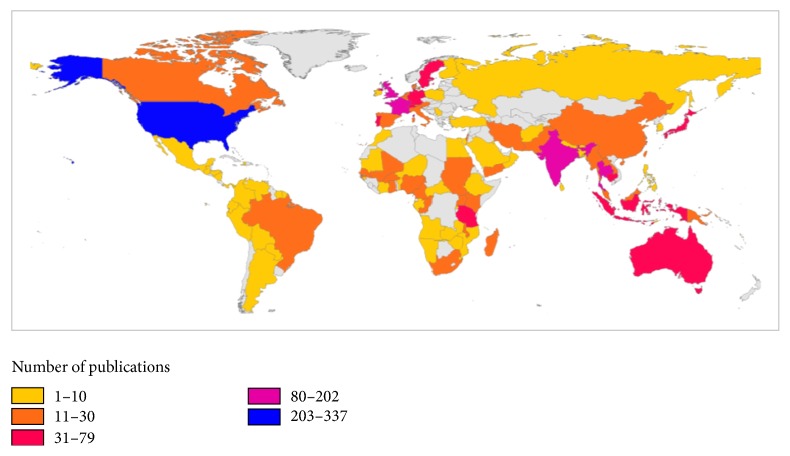
Geographical distribution of retrieved articles in antimalarial drug resistance (2006–2015). Gray regions represent countries where no publications regarding antimalarial drug resistance have been retrieved.

**Figure 5:**
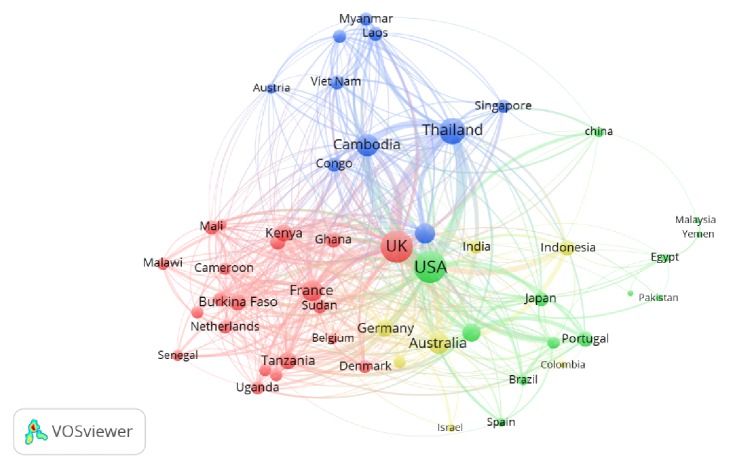
Cluster density visualization map of country coauthorships on AMDR (2006–2015). A minimum of 10 gave a total of 51 items and 4 clusters.

Geographical distribution of retrieved publications was presented in world map using ArcMap 10.1 program (Figure 4). Top countries that participated in publishing documents on AMDR were listed in Table 4. The United States of America (USA) was the most productive country with 337 publications equivalent to one-third of worldwide publications in this field. The USA and the United Kingdom (UK) participated in more than half (55.23%) of worldwide productivity. More than two-thirds of publications by the USA (236, 70.03%) were made by international collaboration with researchers from other countries. All articles published by Cambodian researchers had international authors representing 100% international collaboration (MCP). Furthermore, articles published by Cambodian researchers had the highest number of citations per article when compared with articles published by other countries. Of the top productive countries, two countries were from Mekong subregion, particularly Thailand and Cambodia. Analysis of country coauthorships using VOSviewer showed a map with four clusters (Figure 5, Table 5). Countries in the same cluster have higher collaboration than those distantly located in other clusters. Furthermore, countries with higher number of coauthorships had higher number of articles published on international collaboration.

Top journals in publishing documents about AMDR were listed in Table 6. The Malaria Journal was the most productive journal (136, 13.93%) in this field followed by Antimicrobial Agents and Chemotherapy journal and American Journal of Tropical Medicine and Hygiene. The Proceedings of the National Academy of Sciences (PNAS) had the highest impact factor (9.423) and the highest number of citations per article (49.06). Journal of Infectious Diseases (70.00%) had the highest percentage of highly cited articles followed by PNAS (62.50%). The total number of articles published in the top 10 publishing journals was 450 (46.11%) and the total impact of these articles was 1,704 with an average of 3.79 per article.

Top productive institutions on AMDR were shown in Table 7. The top productive institution was Mahidol University (80, 8.20%) in Thailand. Another institution in the top-ten list was Shoklo Malaria Research Unit in Thailand which was in the 8th position. Three of the top ten institutions active in AMDR research were in Asia, particularly in India and Thailand. Both World Health Organization (WHO) and Centers for Disease Prevention and Control (CDC) were among the top ten productive institutions. Citations per article were the highest for documents published from* Shoklo Malaria Research Center* (107.71) followed by those published by WHO (92.22). For all research institutions/organizations in top-ten list, artemisinin resistance and biomarkers for artemisinin resistance were their major research focus.

Top ten cited articles on AMDR published in the past decade were presented in Table 8. The article “Artemisinin Resistance in* Plasmodium falciparum* Malaria” which received a total of 1350 citations at the time of data analysis (July 15, 2016) was the top cited article. Three articles in the top ten cited list were published in New England Journal of Medicine. Two of the top ten cited articles were published in Science and Nature, and one article was published in The Lancet. Seven articles in the top-ten list were about artemisinins resistance in* Plasmodium falciparum*, one was about CQR, one was about sulfadoxine-pyrimethamine resistance, and the remaining one was about general multidrug resistance.

**Figure 6:**
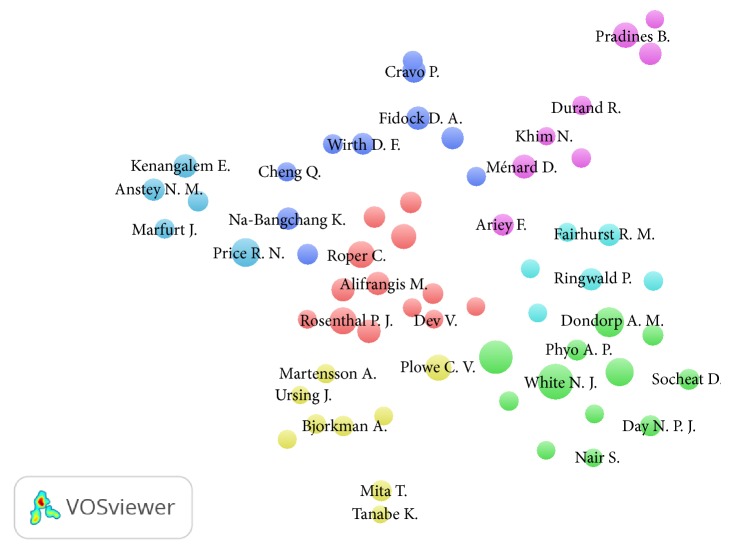
Density visualization map for researchers' coauthorships on AMDR (2006–2015). A minimum of 10 yielded 63 authors.

Authors participating in publications of AMDR with at least 10 documents were shown in the VOSviewer visualization map (Figure 6).  Table 9 lists authors with a minimum of 15 publications and their location in the map. The map contained seven clusters. The most productive authors were clustered together in cluster numbers 1 and 2 mainly.

## 4. Discussion

In this study we aimed to give an overview and an assessment of an emerging important issue regarding antimalarial drug resistance which threatens global efforts to control and eradicate malaria. Although bark of cinchona tree and other related synthetic compounds had been used to treat malaria for centuries, the emergence of resistance to antimalarial drugs is considered relatively recent medical phenomenon. It has been reported that early cases of chloroquine-resistant form of* P. falciparum* appeared in Thailand in the late 1950s. In the 1960s more cases of resistant* P. falciparum *were seen in Southeast Asia followed by the appearance of resistant cases in Sub-Saharan Africa and South America in the 1970s. The spread of chloroquine resistance in the 1970s and 1980s led researcher to develop and introduce new antimalarial drugs to combat the increasing numbers of malaria induced mortality due to antimalarial drug resistance in* P. falciparum *[51–53]. Sulfadoxine-pyrimethamine, an alternative to chloroquine, faced drug-resistant* Plasmodium* species soon after introduction [54]. Unfortunately, most new attempts such as introduction of mefloquine, amodiaquine, and artemisinin faced the same problem of drug resistance with time. The fight against malaria recorded a success upon introduction of insecticide-treated bed nets and indoor residual insecticide spraying [55]. The origin and the emergence of resistance to antimalarial drugs has been developed mainly through genetic mutations which involved chloroquine resistance transporter (PfCRT),* Plasmodium falciparum* multidrug resistance gene-1 (PfMDR), dihydrofolate reductase (DHFR), dihydropteroate synthase (DHPS), and several others [56]. The genetic mutation that led to chloroquine resistance developed independently in Papua New Guinea, certain locations in South America, and Asia which then spread through Southeast Asia and Africa [57–59]. It is believed that spread of resistance to chloroquine did not emerge within infected individuals; rather, it was a spread of emerging mutations due to drug pressure [56]. This hypothesis was tested by removal of drug pressure which led to a decrease in the prevalence of the PfCRT 76T mutation associated with chloroquine resistance [60]. Resistance in* P. falciparum* is complicated by increasing resistance to artemisinin partner drugs such as piperaquine. Molecular markers for drug resistance are currently used for monitoring expected therapeutic outcomes and for directing policy changes towards suitable combination therapies. Markers are available for artemisinin resistance, mefloquine resistance, and recently piperaquine resistance (*plasmepsin 2* and* plasmepsin 3* gene amplifications on chromosome 14) [44, 61–63].

Our study showed that the number of publications on AMDR was fluctuating in the last decade. However, when the number of publications on AMDR was presented for the past five decades, it was apparent that there was an overall increase in the number of publications in the past decade. It was expected that publications on AMDR will decrease with time especially after the introduction of artemisinins as new potent and effective therapy for malaria. However, the emergence of resistance to artemisinins kept the number of publications on AMDR rising with time [64–68]. This new wave of AMDR is accompanied by global concern regarding attaining goals of malaria control in Africa, Asia, and other regions.

The* Global Technical Strategy for Malaria 2016–2030* aimed at reducing incidence, mortality, and resurgence of malaria in endemic countries. This ambitious goal is costly but will save lives and have a cost-effective long term outcome. The emergence of AMDR in general and those pertaining to artemisinin in particular threatens the* Global Technical Strategy for Malaria 2016–2030*. Unfortunately, AMDR reports in the past decade originated from areas suffering from poor health services and depending on international health aids to combat malaria such as some African countries or countries in the Mekong region [69]. The emergence of AMDR is considered a recent phenomenon relative to the long history and extensive use of antimalarials in different parts of the world. Such AMDR were reported in the late 1950s and showed a marked increase and spread in 1970, particularly for those pertaining to chloroquine. This emergence of AMDR was associated with increased malaria rate of death and increased calls by health policy makers and international health organization to discover new antimalarial drugs that are not prone to resistance [51, 70]. In response to this serious threat of AMDR, the* International Centers of Excellence for Malaria Research* (*ICEMRs*) had developed an ICEMR network to monitor AMDR at global level [20].

The global concern on AMDR is manifested in the high h-index value suggesting that there are many readers and citations on the topic. Another indicator for the global concern on AMDR is the top cited articles on AMDR which focused on artemisinin drug resistance in some poor and developing areas like Thailand, Cambodia, and Indonesia. Countries in SEA might be the source of artemisinin drug resistance outbreaks and consequent spread of this resistant to other world regions [71]. The genetic basis of artemisinin drug resistance was common in most areas being investigated in greater Mekong area and is associated with* PfKelch* gene on chromosome 13 (K13) [72]. The potential spread of artemisinin resistance to African countries and other world regions is considered a priority for many international health bodies like WHO. The strategic plan suggested by WHO to prevent the spread or emergence of new geographic spots of artemisinin resistance does not seem to be successful [73]. Understanding the genetic and genomic investigation and the elucidation of molecular markers to AMDR will, hopefully, help in designing new antimalarial drugs. For example, several new compounds are being tested after discovery and understanding of the role of pfcrt in drug resistance [74].

The density visualization maps shed lights on areas of interest on the field of AMDR. The genetic and molecular understanding of AMDR of chloroquine and DHFR inhibitors occupied a central part in the publications on AMDR in the last decade. However. The emerging artemisinins drug resistance also occupied a single large cluster of publications. The publications in the last decade were in the field of molecular biology/genetics of AMDR and characterization of artemisinins drug resistance. These important topics were important in ranking top productive countries. Therefore, developed countries in which molecular and genetic advancement and research are active occupied top ranking positions. Such countries include the USA and the UK. However, countries like Thailand, Cambodia, and India where mainly involved in research pertaining to epidemiology and characterization of the emerging artemisinins drug resistance in Asia region, particularly the Mekong region where malaria is endemic. Publications from Thailand and Cambodia were characterized by high citations per article suggestive of relatively high importance in the field. It seems that all or nearly most of the publications from Thailand and Cambodia came through international research collaboration since this topic is of a global concern and research collaboration in this field is highly needed. Also, the limited resources and expertise of countries in the SER relative to those in Europe and northern American countries made international collaboration a must in order to understand and overcome this serious threat of AMDR to ultimately control the fatal infectious disease of malaria. The research activity on AMDR in Thailand was carried out mainly by two institutions which are presented in the top ten productive institutions along with prestigious organizations and institutions like WHO and CDC.

The retrieved articles discussed various issues that cannot be listed here in detail. However, it is worth commenting on articles that discussed potential causes of AMDR. The WHO recommends artemisinin-based combination therapies (ACTs) for the treatment of malaria to minimize development of artemisinin drug resistance [75, 76]. The ACTs are considered first-line treatment of malaria in most countries and hundreds of millions of ACTs treatment courses were dispensed in the past few years in endemic countries [76]. Therefore drug use without appropriate combination is one mechanism responsible for the development of AMDR [77]. Furthermore, unregulated and irrational use of antimalarial drug use as well as counterfeit and poor quality medicines dispensed in Africa and other parts of the world might be responsible for the spread and development of AMDR [78–80]. Genetic variations of malaria parasites from one region to another are also a potential cause for the development and resistance of AMDR [51, 81, 82].

This study, to the authors' best knowledge, is the first to discuss the AMDR from a bibliometric analysis point of view. However, few limitations pertaining to the study need to be mentioned which have already been mentioned in previous bibliometric studies published by the authors [83–90]. An important limitation is the keywords used which might not be 100% comprehensive and therefore false positive and false negative results are possible. Also, data were retrieved from Scopus and, unfortunately, this does not represent 100% of literature because some journals are not indexed in Scopus. Finally, we analyzed the scientific impact of top ten countries, journals, and institutions and not all data. Despite all this, the authors did their best to validate the data by manual review and tried to give a close overall assessment on ADMR research productivity that hopefully will be a positive addition to the literature on AMDR.

## 5. Conclusion

This study showed an increased interest in the artemisinin drug resistance as well as molecular biology and genetics of AMDR in general. Countries and institutions in the Mekong subregion had a good share of publication on AMDR. International collaboration is of great value and can enhance the quantity and scientific impact of publications on AMDR, particularly in countries with limited resources like the case of some Asian countries. Articles on AMD have been published in prestigious journals with high IF indicative of the global concern and dimension of the AMDR issue.

## Figures and Tables

**Table 1:** Types of retrieved documents on AMDR (2006–2015).

Type of document	Frequency	% *N* = 976
Article	790	80.94
Review	92	9.43
Letter	31	3.18
Note	24	2.46
Short survey	18	1.84
Editorial	9	0.92
Conference paper	6	0.61
Article in press	6	0.61

AMDR: antimalarial drug resistance.

**Table 2:** Growth of annual publications and citations on AMDR (2006–2015).

Year	Total number = 976	%	TC	C/A	CT
2015	109	11.17	608	5.58	21399
2014	105	10.76	1113	10.60	20791
2013	104	10.66	1032	9.92	19678
2012	89	9.12	1834	20.61	18646
2011	98	10.04	1847	18.85	16812
2010	101	10.35	1892	18.73	14965
2009	108	11.07	4635	42.92	13073
2008	72	7.38	2626	36.47	8438
2007	101	10.35	3100	30.69	5812
2006	89	9.12	2712	30.47	2712

AMDR: antimalarial drug resistance; TC: total citations; C/A: citations per article; h-index: Hirsh index; CT: cumulative citations.

**Table 3:** Most frequent terms in title/abstract of publications on AMDR (2006–2015) using VOSviewer technique.

Terms related to drug resistance, gene mutations, or countries	Number of occurrences
*Cluster # 1 (red) = 138 items*	
Chloroquine resistance transporter	20
Chloroquine-resistant	15
Chloroquine-resistant malaria	21
Chloroquine-resistant *P. falciparum*	11
Chloroquine-resistant parasites	12
Chloroquine-resistant *Plasmodium falciparum*	26
Chloroquine-resistant strain	20
CQR (chloroquine resistance)	38
pfcrt mutation	15
Transporter	38
Protein	92
Phenotype	62

*Cluster # 2 (green) = 123 items*	
Antifolate drug resistance	25
dhfr gene	18
dhps gene	33
dhps mutation	18
pfdhps gene	20
Pyrimethamine resistance	38
pfcrt gene	39
pfmdr1 gene	35

*Cluster # 3 (blue) = 89 items*	
Artemisinin resistance	90
Artemisinin-resistant malaria	17
Sub-Saharan Africa	23
Thai-Myanmar border	10
Papua	15
Asia	81

AMDR: antimalarial drug resistance.

**Table 4:** Top ten productive countries, scientific impact, and international collaboration on AMDR (2006–2015).

Rank	Country	Frequency *N* = 976	TC	C/A	h-index	CC	SCP	MCP
1st	United States	337 (34.53)	9498	28.18	50	73	101 (29.97)	236 (70.03)
2nd	United Kingdom	202 (20.70)	8702	43.08	48	69	15 (7.43)	187 (92.57)
3rd	Thailand	129 (13.22)	6736	52.22	39	51	30 (23.26)	99 (76.74)
4th	France	90 (9.22)	2463	27.37	28	61	19 (21.11)	71 (78.89)
5th	India	89 (9.12)	1280	14.38	18	30	65 (73.03)	24 (26.97)
6th	Australia	79 (8.09)	3054	38.66	28	45	10 (12.66)	69 (87.34)
7th	Cambodia	49 (5.02)	4645	94.80	27	45	0 (0.00)	49 (100.00)
7th	Switzerland	49 (5.02)	3143	64.14	22	45	0 (0.00)	49 (100.00)
9th	Germany	46 (4.71)	1255	27.28	25	54	8 (17.39)	41 (89.13)
10th	Portugal	42 (4.30)	970	23.10	16	29	3 (7.14)	39 (92.86)

AMDR: antimalarial drug resistance; *N*: total number of publications; TC: total citations; h-index: Hirsch index; CC: cumulative citations; SCP: single country publications; MCP: multiple country publications.

**Table 5:** Country coauthorship as retrieved by VOSviewer. A minimum of 10 gave a total of 51 items and 4 clusters.

Cluster number	Items (number of country coauthorships)
Cluster # 1 (red) 21 items	Belgium (31), Burkina Faso (94), Cameroon (53), Denmark (61), Ethiopia (47), France (182), Gambia (67), Ghana (79), Italy (29), Kenya (112), Madagascar (45), Malawi (47), Mali (76), Netherlands (58), Nigeria (80), Senegal (42), South Africa (47), Sudan (69), Tanzania (121), Uganda (63), United Kingdom (518).
Cluster # 2 (green) 13 items	Brazil (35), China (33), Egypt (17), Iran (8), Japan (66), Malaysia (10), Pakistan (13), Papua New Guinea (53), Portugal (77), Spain (27), Sweden (125), USA (529), Yemen (10).
Cluster # 3 (blue) 10 items	Austria (32), Bangladesh (60), Cambodia (203), Congo (70), Laos (76), Myanmar (56), Singapore (56), Switzerland (158), Thailand (312), Vietnam (69).
Cluster # 4 (yellowish green) 7 items	Australia (197), Canada (47), Colombia (15), Germany (109), India (62), Indonesia (86), Israel (16).

**Table 6:** Top ten journals in publishing articles on AMDR (2006–2015).

Rank	Journal	Frequency (%) *N* = 976	TC	h-index	C/A	HC (%)	IF	Total IF
1st	Malaria Journal	136 (13.93)	1860	22	13.68	29 (21.32)	3.079	418.744
2nd	Antimicrobial Agents and Chemotherapy	96 (9.84)	2017	27	21.01	41 (42.71)	3.34	320.64
3rd	American Journal of Tropical Medicine and Hygiene	56 (5.74)	1124	19	20.07	19 (33.93)	2.699	151.144
4th	Plos One	38 (3.89)	733	16	19.29	13 (34.21)	3.54	134.52
5th	Journal of Infectious Diseases	30 (3.07)	997	21	33.23	21 (70.00)	6.344	190.32
6th	Acta Tropica	26 (2.66)	358	12	13.77	7 (26.92)	2.380	61.88
7th	Infection Genetics and Evolution	21 (2.15)	285	9	13.57	5 (23.81)	2.591	54.411
8th	PNAS	16 (1.64)	785	12	49.06	10 (62.50)	9.423	150.768
8th	Trends in Parasitology	16 (1.64)	242	9	15.13	4 (25.00)	7.295	116.72
10th	Emerging Infectious Diseases	15 (1.54)	229	9	15.27	3 (20.00)	6.99	104.85

	*Total*	*450 (46.11%)*						*1,704*

PNAS: Proceedings of the National Academy of Sciences of the United States of America; AMDR: antimalarial drug resistance; *N*: total number of publications; TC: total citations; h-index: Hirsch index; C/A: citations per article; IF: impact factor.

**Table 7:** Top ten productive institutions in publishing articles on AMDR (2006–2015).

Rank	Institution (affiliation)	Country	Frequency (%)	TC	C/A	h-index	HC (%)
1st	Mahidol University	Thailand	80 (8.20)	5197	64.96	34	38 (47.50)
2nd	London School of Hygiene & Tropical Medicine	UK	62 (6.35)	2033	32.79	25	21 (33.87)
3rd	Oxford University (Nuffield Department of Clinical Medicine)	UK	45 (4.61)	1471	32.69	20	15 (33.33)
4th	National Institute of Allergy and Infectious Diseases	USA	39 (4.00)	2247	57.62	23	21 (53.85)
5th	National Institute of Malaria Research India	India	37 (3.79)	2951	79.76	19	15 (40.54)
6th	Organisation Mondiale de la Sante	WHO	32 (3.28)	2951	92.22	19	15 (46.88)
7th	Centers for Disease Control and Prevention	USA	31 (3.18)	877	28.29	16	9 (29.03)
7th	Menzies School Of Health Research	Australia	29 (2.97)	1854	63.93	20	16 (55.17)
9th	Shoklo Malaria Research Unit	Thailand	28 (2.87)	3016	107.71	20	19 (67.86)
10th	University of California, San Francisco	USA	26 (2.66)	691	26.58	15	10 (38.46)

TC: total citations; C/A: citations per article; h-index: Hirsch index; HC (%): percentage of articles with high citations.

**Table 8:** Top cited articles on AMDR (2006–2015).

Rank	Authors	Title	Source title	Number of citations
1st	Dondorp et al. [41]	“Artemisinin Resistance in *Plasmodium falciparum* Malaria”	New England Journal of Medicine	1350
2nd	Noedl et al. [42]	“Evidence of Artemisinin-Resistant Malaria in Western Cambodia”	New England Journal of Medicine	688
3rd	Phyo et al. [43]	“Emergence of Artemisinin-Resistant Malaria on the Western Border of Thailand: A Longitudinal Study”	The Lancet	354
4th	Ariey et al. [44]	“A molecular Marker of Artemisinin-Resistant *Plasmodium falciparum* Malaria”	Nature	319
5th	Tjitra et al. [45]	“Multidrug-Resistant *Plasmodium vivax* Associated with Severe and Fatal Malaria: A Prospective Study in Papua, Indonesia”	PLoS Medicine	301
6th	Ashley et al. [46]	“Spread of Artemisinin Resistance in *Plasmodium falciparum* Malaria”	New England Journal of Medicine	263
7th	Price et al. [47]	“New Developments in *Plasmodium vivax* Malaria: Severe Disease and the Rise of Chloroquine Resistance”	Current Opinion in Infectious Diseases	197
8th	Cheeseman et al. [48]	“A Major Genome Region Underlying Artemisinin Resistance in Malaria”	Science	172
9th	Ter Kuile et al. [49]	“Effect of Sulfadoxine-Pyrimethamine Resistance on the Efficacy of Intermittent Preventive Therapy for Malaria Control during Pregnancy: A Systematic Review”	Journal of the American Medical Association	170
10th	Price et al. [50]	“Molecular and Pharmacological Determinants of the Therapeutic Response to Artemether-Lumefantrine in Multidrug-Resistant *Plasmodium falciparum* Malaria”	Clinical Infectious Diseases	161

**Table 9:** List of authors, number of coauthorships, and location in cluster as retrieved from VOSviewer. Researchers with a minimum of 15 documents on AMDR (2006–2015) were shown.

Author	Number of publications	Number of coauthorships	Cluster
Nosten, F.	35	148	2
White, N. J.	28	186	2
Imwong, M.	25	131	2
Price, R. N.	25	88	7
Roper, C.	23	15	1
Plowe, C. V.	21	74	4
Pradines, B.	21	28	5
Dondorp, A. M.	20	149	2
Rosenthal, P. J.	20	22	1
Udhayakumar, V.	19	31	1
Alifrangis, M.	17	8	1
Kenangalem, E.	17	55	7
Rogier, C.	17	27	5
Meshnick, S. R.	16	35	1
Ménard, D.	24	41	5
Sutherland, C. J.	16	22	1
Anstey, N. M.	15	51	7
Fairhurst, R. M.	15	81	6
Fidock, D. A.	15	14	3
Na-Bangchang, K.	15	3	3
Ringwald, P.	15	70	6
Roepe, P. D.	15	5	3
Wirth, D. F.	15	6	3

## Abbreviations

UNICEF: United Nations Children's Emergency Fund
WHO: World Health Organization
CDC: Centers for Disease Preventions and Control
AMDR: Antimalarial drug resistance.

## Data Access

All data present in this article can be retrieved from Scopus using keywords listed in the methodology.
